# Mathematics Anxiety, Working Memory, and Mathematics Performance in Secondary-School Children

**DOI:** 10.3389/fpsyg.2016.00042

**Published:** 2016-02-02

**Authors:** Maria C. Passolunghi, Sara Caviola, Ruggero De Agostini, Chiara Perin, Irene C. Mammarella

**Affiliations:** ^1^Department of Psychology, University of TriesteTrieste, Italy; ^2^Department of Developmental and Social Psychology, University of PadovaPadova, Italy

**Keywords:** mathematics anxiety, short-term memory, working memory, inhibitory control

## Abstract

Mathematics anxiety (MA) has been defined as “a feeling of tension and anxiety that interferes with the manipulation of numbers and the solving of math problems in a wide variety of ordinary life and academic situations.” Previous studies have suggested that a notable proportion of children in primary and secondary school suffer from MA, which is negatively correlated with calculation skills. The processing efficiency and attentional control theories suggest that working memory (WM) also plays an important part in such anxious feelings. The present study aimed to analyze the academic achievement and cognitive profiles of students with high math anxiety (HMA) and low math anxiety (LMA). Specifically, 32 students with HMA and 34 with LMA matched for age, gender, generalized anxiety, and vocabulary attending sixth to eighth grades were selected from a larger sample. The two groups were tested on reading decoding, reading comprehension, mathematics achievement, and on verbal short-term memory and WM. Our findings showed that HMA students were weak in several measures of mathematics achievement, but not in reading and writing skills, and that students with HMA reported lower scores on short-term memory and WM performances (with associated difficulties in inhibiting irrelevant information) than children with LMA. In addition, a logistic regression showed that weaknesses in inhibitory control and fact retrieval were the strongest variables for classifying children as having HMA or LMA.

## Introduction

Mathematical difficulties may be seen not only in children with specific mathematical learning disorders, but also in those with emotional issues, such as mathematics anxiety (MA) (Ashcraft and Kirk, 2001; Maloney and Beilock, 2012; Vukovic et al., 2013). Previous studies have shown that individuals with MA have increasing difficulty the greater the demand of the mathematical problem (Ashcraft and Moore, 2009). A negative loop is generated in which these individuals often perform badly in standardized math tests (Hembree, 1990; Ashcraft and Krause, 2007), avoid arithmetic courses (Hembree, 1990; Ashcraft and Moore, 2009), and develop negative beliefs regarding their own math abilities (Lent et al., 1991; Ashcraft and Kirk, 2001), thus experiencing even more MA and avoidance. Given the long-term damaging effects of MA, it is important to understand how MA affects mathematics achievement.

Mathematics builds on several cognitive abilities (Passolunghi et al., 2008; Krajewski and Schneider, 2009; Geary, 2011) implemented by an extensive neural network in the brain (Goswami and Szűcs, 2011; Fias et al., 2013), and influenced by emotional aspects (such as feelings of apprehension, dislike, tension, worry, frustration, and fear) experienced when performing mathematical tasks, which goes by the name of mathematics anxiety. On the relationship between MA and cognitive processes, previous studies have shown that individuals with a limited working memory (WM) capacity may experience difficulty in regulating their anxiety levels (Hofmann et al., 2011), and anxiety/worry may reduce their WM resources (Eysenck et al., 2007; Mammarella et al., 2015). It is common knowledge that cognitive skills such as WM, processing speed, attention and inhibition are important in the setting of mathematical learning difficulties (Fletcher et al., 2007). The cognitive consequences of MA have also been characterized in several studies, which have associated MA with an impaired WM and attention capacity (e.g., Ramirez et al., 2013).

Theories on processing efficiency and attentional control suggest an important role for WM in regulating cognitive performances (Eysenck and Calvo, 1992; Richards and Gross, 2000; Eysenck et al., 2007). According to processing efficiency theory and attentional control theory (ACT), worrying (which is the cognitive component of anxiety) is believed to demand processing competence, thereby reducing the WM capacity available for other tasks (Eysenck and Calvo, 1992; Ashcraft and Kirk, 2001; Derakshan and Eysenck, 2009; Eysenck and Derakshan, 2011). In particular, the ACT approach (Eysenck and Calvo, 1992) assumes that anxiety interferes with the efficient functioning of the goal-directed attentional system, as well as reducing attentional control; in other words, anxiety raises an individual's attention to threat-related stimuli. According to the ACT approach, the negative effects of anxiety on processing efficiency would therefore stem from two executive functions involving attentional control: inhibition and shifting (Eysenck et al., 2007). That does not mean that the quality of an individual's performance (as usually assessed by means of standard behavioral measures such as response accuracy) is necessarily impaired, especially if their anxiety prompts the use of compensatory strategies (e.g., more effort, or a greater use of processing resources).

The complexity of the interaction between cognition and emotion also depends on the difficulty of the arithmetical tasks proposed. For instance, in investigating the effects of WM on emotion regulation, different arithmetical tasks have been used to manipulate the load on WM (Van Dillen and Koole, 2009; Van Dillen et al., 2009; Kanske et al., 2011), and math-specific anxiety has been associated with a reduced WM capacity and with a slow and inaccurate handling of arithmetical problems (Ashcraft and Faust, 1994; Ashcraft and Kirk, 2001; Mattarella-Micke et al., 2011; Suárez-Pellicioni et al., 2013). Interactions between negative emotion and WM capacity have also been shown to affect more complex math problem solving and reasoning abilities (Owens et al., 2014). In particular, Owens et al. (2014) found that high levels of anxiety negatively affected math reasoning in individuals with relatively small digit and spatial spans, whereas they positively affected reasoning ability in those with a high WM span.

Surprisingly little is known about the specific detrimental effect of MA on academic achievement in middle school students. Most previous studies were conducted on young adults, or children in the early stage of math learning (Wu et al., 2012; Ramirez et al., 2013), while few studies have included participants of middle school by studying the relationship between MA and algebraic problem solving in 14 years-old (Trezise and Reeve, 2014, 2015). The present study, aimed to investigate the relationship between MA and mathematical achievement in a group of middle school students (in grades six to eight) in order to fill the gap of the previous literature. In addition, we aimed to look into: how specifically the detrimental effect of MA concerned math achievement, but not reading and writing achievement; and the relationship between MA and WM, also considering the role of inhibitory processes at this particular developmental age.

The present study was therefore designed to investigate whether (a) different patterns of mathematical abilities emerge in two groups of middle school students selected on the basis of their level of MA (but matched for general anxiety); (b) students with high or low levels of MA were also impaired in different areas of academic achievement, such as reading decoding, reading comprehension and writing abilities; (c) the two groups of students performed differently in measures of verbal short-term memory and WM; (d) different levels of MA were associated with the ability to inhibit irrelevant information. Finally, (e) in the present research, the variables able to identify children with high and low MA were also analyzed. To investigate these issues, we used a similar paradigm to the one employed by Passolunghi (2011) and Passolunghi and Siegel (2001, 2004), with an exception regarding the groups' selection. In a previous study, Passolunghi (2011) examined emotional and cognitive factors in groups of children with and without mathematical learning disabilities (MLD). In the present research, we focused instead on children with high or low levels of MA and investigated their profiles in different areas of cognitive and academic achievement. Given the specificity of the worrying thoughts, we hypothesized that children with HMA would show specific impairments in most of the mathematical tasks proposed, but no differences in reading and writing tasks. In addition, we expected children with high levels of math anxiety (HMA) to be more impaired in WM and inhibitory control than children with low levels of math anxiety (LMA), since task-irrelevant thoughts would disrupt the former's performance because they would reduce the pool of resources available (Ashcraft and Kirk, 2001; Ashcraft and Krause, 2007).

## Methods

### Screening phase

The initial screening phase involved a sample of 135 children (64 Males, 71 Females) aged 11–13 years (*M* = 155.22 months; *SD* = 12.78), comprising 47 children in sixth grade, 49 in seventh, and 39 in eighth grade, all attending state schools in Northern Italy and coming from families of middle socioeconomic status.

Parental consent was obtained for all the children prior to testing. Children with intellectual disabilities, learning disabilities, or an inadequate command of the Italian language were excluded from the study. Students participating in the screening phase were tested collectively in their classrooms during regular class hours.

Children with a HMA or LMA were identified on the basis of their performance and anxiety levels recorded by means of screening tests. Their level of MA was measured with an adaptation of the Abbreviated Math Anxiety Scale (AMAS, Hopko et al., 2003), while their general level of anxiety was tested using the Revised Children's Manifest Anxiety Scale—2nd Edition (RCMAS-2, Reynolds and Richmond, 2012). To control for general cognitive skills, we tested the students' vocabulary (PMA-Verbal meaning, Thurstone and Thurstone, 1981)^1^.

The inclusion criteria for LMA children were as follows: (a) average scores for MA; (b) average scores for general anxiety; and (c) average scores for PMA-Verbal meaning. The inclusion criteria for the HMA group were: (a) scores higher than 1 SD for MA; (b) average scores for general anxiety; and (c) average scores for PMA-Verbal meaning. From the initial sample, only 36 children had scores higher than 1 SD on MA. Four children were excluded: three did not have average scores on the PMA Verbal meaning, and one showed scores higher than 1 SD on general anxiety. Thus, our final group was composed of 32 HMA children.

### Participants

Our final sample consisted of 34 LMA children (21 females), including 9 sixth-, 16 seventh-, and 9 eighth-graders, and 32 HMA children (22 females), with 11 of them in sixth, 12 in seventh, and 9 in eighth grade. The groups' characteristics and appropriate statistics are shown in Table 1.

**Table 1:** **Descriptive (M, means; SD, standard deviations) and one-way ANOVAs of the comparison between children with low (LMA) and high levels of math anxiety (HMA)**.

	**LMA M(SD)**	**HMA M(SD)**	***F*_(1, 64)_ =**	***p***	**η^2^**
Age in months	155.62 (10.56)	156.06 (14.46)	0.02	0.881	>0.001
**GENERAL COGNITIVE AND EMOTIONAL PROFILES**					
PMA—vocabulary	14.50 (7.86)	13.22 (7.47)	0.46	0.502	0.007
RCMAS -2 (T scores)	53.26 (7.11)	55.06 (9.04)	0.81	0.370	0.013
AMAS	19.56 (3.03)	29.16 (3.45)	144.91	0.001	0.690
**ACADEMIC ACHIEVEMENT (Z SCORES)**					
**Mathematical proficiency, AC-MT battery**					
Written calculation	0.04 (0.82)	−0.66 (0.93)	10.67	0.002	0.143
Magnitude judgment	0.37 (0.88)	−0.12 (1.05)	4.31	0.040	0.063
Place-value comprehension	−0.07 (0.99)	−0.50 (1.10)	2.84	0.090	0.042
Logical reasoning	−0.09 (0.93)	−0.66 (1.09)	5.17	0.030	0.075
Approximate calculation	−0.16 (0.86)	−0.36 (0.98)	0.83	0.370	0.013
Fact retrieval	0.01 (0.83)	−0.67 (0.96)	9.20	0.003	0.126
**Reading comprehension**					
Comprehension	−0.14 (0.71)	−0.41 (0.98)	1.67	0.210	0.025
**Word reading and writing**					
Reading speed	0.40 (1.29)	.71 (0.94)	1.23	0.270	0.019
Reading accuracy	0.37 (1.13)	0.61 (1.01)	0.78	0.380	0.012
Writing accuracy	0.21 (2.61)	−0.13 (0.94)	0.45	0.500	0.007
**Working memory measures**					
STM—Number of words	23.35 (4.22)	19.63 (4.35)	6.35	0.014	0.090
LST—Number of words	24.12 (4.28)	21.78 (4.26)	4.94	0.030	0.072
LST—Intrusion errors	1.88 (1.45)	3.47 (2.51)	10.01	0.002	0.135

*AMAS, Abbreviated Math Anxiety Scale (Hopko et al., 2003); RCMAS, Revised Children's Manifest Anxiety Scale -2nd Edition (Reynolds and Richmond, 2012); STM, Short-Term Memory; LST, Listening Span Test*.

In the second phase, the two groups of children were tested during two different sessions to assess any differences between their academic and cognitive aspects by means of: (a) a collective session during which the students completed tests on their mathematical proficiency and reading comprehension; and (b) individual sessions in which they were assessed on their word reading and writing abilities, and their working memory.

## Materials

### Screening phase

*The Abbreviated Math Anxiety Scale* (AMAS; derived by Hopko et al., 2003) is a self-report MA questionnaire adapted to middle-school students^2^. With 9 items, it is the shortest valid MA scale, but in the original version it has been shown to be as effective as the longer Maths Anxiety Rating Scale (MARS; Hopko, 2003) (e.g., internal consistency: Cronbach's α = 0.90; 2-week test-retest reliability: *r* = 0.85; convergent validity of AMAS and MARSR *r* = 0.85). Using a 5-point Likert scale, participants indicate how much anxiety (e.g., 1 = low anxiety; 5 = high anxiety) they would feel during certain situations involving maths. In our adaptation to middle-school students two items were slightly modified (item 1: “Having to use the tables and math formulas in the back of a math book”; item 6: “Being given an assignment of many difficult math exercises due to the next class meeting”). The Cronbach's α calculated on our sample was = 0.81.

*The Revised Children's Manifest Anxiety Scale: Second Edition* (RCMAS-2; Reynolds and Richmond, 2012) is a self-report questionnaire used to identify the source and level of GA in children from 6 to 19 years old. It consists of 49 items with a simple yes/no response format and is divided into 5 different scales: physiological anxiety, worries, social anxiety, defensiveness and total anxiety (internal consistency: physiological anxiety Cronbach's α = 0.68; worries α = 0.80; social anxiety α = 0.78; defensiveness α = 0.70; total anxiety α = 0.89).

*Verbal Meaning, Primary Mental Abilities* (PMA, Thurstone and Thurstone, 1981). The verbal meaning subtest comprises 51 trials in which participants are given a target word and are required to choose among five alternative words which one has the same meaning of the target. The final score is given by the sum of correct responses minus incorrect responses The test-retest reliability is *r* = 0.92.

### Academic achievement measures

#### Mathematics achievement

Mathematical abilities were assessed using the AC-MT 11–16 standardized mathematics test (Cornoldi and Cazzola, 2004) designed for sixth- to eighth-graders. This test assesses calculation procedures and number comprehension by means of a set of paper-and-pencil tasks that can be grouped into two areas: “written calculation” and “number knowledge.” In the former, participants have to solve eight written multi-digit calculations (two additions, two subtractions, two multiplications and two divisions). The latter contains tasks demand involve number magnitude judgments, place-value (i.e., syntax) comprehension, logical reasoning, approximate calculations, and fact retrieval (i.e., solve 32 simple calculations in a time limit of 2 min). The test re-test reliability is *r* = 0.83. Z-scores were calculated on the basis of the normative sample according to grades.

### Reading and writing achievement

#### Reading comprehension

This task was derived from the standardized MT battery (Cornoldi et al., 2010). It focuses mainly on the student's ability to find appropriate information within a text to answer a set of comprehension questions, enabling comprehension to be considered separately from the contribution of decoding and memory of the text (Cornoldi and Oakhill, 1996). Participants are asked to silently read a passage and then answer some questions related to the text. They are given an unlimited amount of time to complete the task and they are allowed to consult the text as often as they wish. (Cronbach's α = 0.77). Z-scores were calculated on the basis of the normative sample according to grades.

#### Word reading and writing

These tasks are subtests of a battery specifically for assessing developmental dyslexia and dysorthographia (Sartori et al., 2007). The battery has an adequate reliability (e.g., mean test-retest coefficients are 0.77 for speed and 0.56 for accuracy). In the Word reading task participants are asked to read four lists of isolated words aloud and as accurately and rapidly as possible. The material varies in frequency and concreteness, starting with a list of very common and concrete words, followed by lists of words of decreasing frequency and concreteness. Reading speed is calculated by dividing the number of syllables read by the time (in seconds) taken to read them. Accuracy corresponds to the number of words read incorrectly. The Word writing task involves participants writing lists of words. The materials are presented aloud by the experimenter who dictates the words at a constant rhythm (about one word every 3 s). The score is represented by the number of words that are written incorrectly. Z-scores were calculated on the basis of the normative sample according to grades.

### Verbal short-term and working memory tasks

#### Verbal short-term memory (STM)

To assess the student's short-term memory ability, we used the Word Span Forward task (Passolunghi and Siegel, 2004), which involves the passive storage and recall of verbal information (Swanson, 1993; Cornoldi and Vecchi, 2000). The task consists in the presentation of lists of words of increasing length (from 3 to 8 words) and the participant has to remember the words in the same order as they were presented. Two trials are run for each length of word list. There is no time limit for recalling the words in the same, forward order. The raw score is the number of words correctly remembered throughout the testing session.

#### Listening span task (LST)

To analyze working memory we used an adaptation of the listening span test devised by Daneman and Carpenter (1980). This test engages the participant in a dual task: the child has to judge whether a sentence is true or false and also retain the last word in the sentence. The sentences are arranged into sets of different length, from 2 to 5 sentences per set, with two sentences in the first sets and increasing the number of sentences in later sets. At the end of each set of sentences, immediately after saying whether a sentence was true or false, participants are asked to recall the last word in each sentence (in the same order as they were presented) and to be careful to avoid naming non-final words. Two scores are obtained from this recall task: one for the raw number of words recalled correctly, and one for the number of non-target words erroneously recalled (intrusion errors). The latter score is considered a measure of cognitive inhibition processes (De Beni et al., 1998; Passolunghi and Siegel, 2001, 2004).

### Procedure

The experimental procedure described here was in accordance with the Declaration of Helsinki (Sixth revision, 2008). After assigning them to one or other group the children were tested during two different sessions lasting approximately 30 min each. They were first tested collectively with the AC-MT 11–14 standardized arithmetic battery (Cornoldi and Cazzola, 2004) and the reading comprehension task (MT battery, Cornoldi et al., 2010). Then in a second session, the children were tested individually in a quiet room away from their classroom, where the word reading and writing subtests (Sartori et al., 2007) and the STM and WM tasks were administered.

## Results

First, we compared LMA and HMA groups in the screening measures, academic and WM tasks to identify any statistically significant differences. Table 1 summarizes the performance of the LMA and HMA children in all the tests administered. All statistical analyses (see Table 1) refer to the comparison of the two groups using one-way ANOVA; the effect sizes ($\eta_{p}^{2}$) are also reported.

### Screening phase

LMA and HMA groups did not differ in terms of PMA-Vocabulary or generalized anxiety (*p* > 0.37); however, the HMA group showed much higher levels of MA than the LMA group *F*_(1, 64)_ = 144.91; *p* < 0.0001. In addition, the two groups were matched for age *F*_(1, 64)_ < 1, and gender χ^2^ (1, *N* = 66) = 0.35; *p* < 0.55.

### Academic achievement tasks

For mathematical proficiency, as expected, we found significant differences in the written calculation task, and in some tests in the “number knowledge” part of the AC-MT battery. In particular, the two groups showed significant differences in four tests—Written calculation, Magnitude judgment, Logical reasoning and Fact retrieval—for all of which the LMA group outperformed the HMA group by more than 0.5 standard deviation.

When the groups were compared on the other academic measures, we found no significant differences for reading comprehension accuracy or the word reading and writing measures (see Table 1). The mean z-scores for reading comprehension and word reading indicate that LMA children performed slightly better than HMA children. The opposite pattern (falling short, here again, of statistical significance) emerged for the writing tasks: children with HMA were slightly more accurate in word writing than those with LMA.

### Working memory tasks

As shown in Table 1, significant differences were found between the two groups in all the measures of verbal short-term and working memory. Individuals with HMA recalled significantly fewer words than LMA children in the STM and LST and made more intrusion errors in the LST.

### Logistic regression

To see which tasks could discriminate between individuals with HMA and those with LMA, we conducted a likelihood-ratio logistic regression analysis using the Wald method. Logistic regression applies maximum likelihood estimation after transforming the dependent into a logit variable (the natural log of the odds of the dependent variable occurring or not).

The model created included one dependent—or criterion—variable of the LMA or HMA groups and two independent—or predictor—variables found significant (i.e., fact retrieval, *Wald* χ^2^(1) = 7.16, *p* = 0.007, and intrusion errors in the LST, *Wald* χ^2^(1) = 7.00, *p* = 0.008, see Table 2), *R*^2^_(*Cox&Snell*)_ = 0.24.

**Table 2:** **Percentage of individuals correctly identified by the model**.

**Observed**	**Predicted**		
	**Group**		**Percentage identified correctly**
	**LMA**	**HMA**	
LMA	27	7	79.4
HMA	10	22	68.8
Overall			74.2

*LMA, low mathematical anxiety; HMA, high mathematical anxiety*.

As shown in Table 2, the two predictor variables were able to identify 79.4% of the LMA children and 68.8% of the HMA children. In other words, the probability that a child of the LMA was correctly identified using the predictor variables is 79.4%; whereas the probability that a child of the HMA group was correctly identified using the predictor variables is 68.8%. A Hosmer-Lemeshow test was conducted to examine the goodness of fit of our logistic model against actual outcomes. The Hosmer-Lemeshow test yielded a χ^2^(7) = 2.72, *p* = 0.91, indicating a good fit.

## Discussion

The main aim of this study was to analyze the academic achievement and cognitive profiles of children with HMA and LMA in middle school students, given that most previous studies were conducted on young adults, or children in the early stage of math learning. For this reason, we selected children in sixth to eighth grade who had HMA but not generalized anxiety, and a group with LMA matched for age, gender, generalized anxiety, and vocabulary. The children were tested on reading decoding, reading comprehension, mathematics achievement, and also on verbal short-term and working memory. We thus analyzed whether children with HMA were only weak in mathematics, but not in reading and writing, and whether these children with HMA had a lower WM performance (and associated difficulty with inhibiting irrelevant information) than children with LMA.

Concerning their academic achievement, children with HMA performed less well than those with LMA in all mathematical tasks except for the approximate calculation subtest, whereas the two groups did not differ on reading decoding, reading comprehension and word dictation. In agreement with previous findings (Hembree, 1990; Ashcraft and Krause, 2007), the present study thus confirmed that high levels of mathematics anxiety coincide with a high likelihood of a poor academic performance, but only in mathematics. It is worth noting that the negative relationship between math anxiety and achievement does not produce a general impairment in all achievement tasks, in fact, only on mathematics achievement children with HMA were specifically impaired (see also Ashcraft and Moore, 2009).

As for the cognitive profile of children with high and low levels of MA, our results showed that children with HMA performed less well on both verbal STM and WM tasks. Unlike several previous studies, we found our HMA children impaired in a verbal STM task involved no digits or computations (Ashcraft and Kirk, 2001; Mammarella et al., 2015). Such an impairment on verbal WM tasks had already been reported (Ashcraft and Kirk, 2001; Eysenck et al., 2007; Ramirez et al., 2013; Mammarella et al., 2015), and suggests that anxiety may reduce verbal WM resources. In particular, Ramirez et al. (2013) who studied children attending first and second grades revealed that children with high WM showed a pronounced negative relation between math anxiety and math achievement, hence the present study—using a different approach—extend the negative relation among MA, WM, and math achievement to older students. In another recent, study testing middle school students with high and low MA, Mammarella et al. (2015) showed that students with HMA with and without math difficulties performed worse than students with typical development in a verbal WM task (i.e., backward words span task), in agreement with the present findings. However, the backward words span task does not allow to analyze the ability to inhibit irrelevant information in WM (Passolunghi and Siegel, 2001) and that is why we chose a typical dual-span task to investigate WM processes in the present study. Our children with HMA, in fact, made more intrusion errors, thus showing to be unable to inhibit irrelevant information in WM, than the children with LMA.

It is worth noting that our HMA group revealed not only weaknesses in inhibiting irrelevant information in WM, but also specifically failed in mathematical tasks and not in verbal (reading and writing) tasks. This pattern of results is consistent with the ACT model proposed by Eysenck and Calvo (1992), confirming that math anxiety interferes with the efficient functioning of the goal-directed attentional system, reducing attentional control specifically on math-related tasks. This conclusion is strengthened by the results of our logistic regression, in which only intrusion errors in the LST and fact retrieval emerged as significant predictors of math anxiety: these two measures correctly identified around 79% of children with LMA and 69% of children with HMA. It is worth noting that our fact retrieval task involved producing the correct answer for simple calculations under time constraints, and children with HMA may be at a greater disadvantage when under pressure to respond promptly. Faust et al. reported (1996) finding no differences relating to math anxiety in their sample's accuracy on untimed paper-and-pencil tests, but the same stimuli generated anxiety effects in the task with time constraints.

A possible limitation of our study lies in our choice of STM and WM tasks. In fact, only the verbal component was investigated, so further studies should compare children with HMA and LMA on both verbal and visuospatial STM and WM tasks. Gender-related differences were not examined in the present study either. When Devine et al. (2012) studied a large sample of children of middle school, they found that girls and boys performed equally well in math, but girls experienced more math anxiety than boys. In our sample too, around 70% of the participants with HMA were girls. Further studies should nonetheless analyze gender-related differences in mathematics anxiety, WM and academic achievement in more depth. In addition, the present study was not able to disentangle the direction of the relationship among MA, WM and mathematics performances, therefore, further research should analyze whether WM (and in particular difficulties in inhibiting irrelevant information) mediates the effects on mathematics performances and the relationship between MA and arithmetic achievement. Finally, ours was a cross-sectional study, whereas a longitudinal study would be able to generate information on how the relationship between mathematics anxiety, WM and math performance evolve over time.

The present study has some implications for educators. First, weak math abilities and low WM capacity may be seen as risk factors for math anxiety. Math anxiety seems to have a straightforward influence on cognitive processing, not only impairing WM, but also making children with HMA perform less well than children with LMA in mathematical tasks. Being aware of which middle school students experience high levels of MA could help teachers try to avoid the vicious circle triggered when anxiety leads to the avoidance of situations involving math tasks. For example, given that pressure may affect math performances of students with HMA, teachers should avoid time constraints for students showing high levels of MA; in addition, teachers should provide students with feedback about the correctness of their responses, since previous findings showed that worries tent to increase after negative feedback, and decrease after positive feedback (Daniels and Larson, 2001).

In conclusion, the present study showed that middle school students with HMA are at greater risk than those with LMA of performing poorly in mathematics achievement measures. HMA students also performed less well in verbal STM and WM tasks, and were less able to inhibit irrelevant information. Finally, measures of inhibitory control and fact retrieval emerged as the best predictors for identifying children with high or low levels of MA.

## Author contributions

All authors listed, have made substantial, direct and intellectual contribution to the work, and approved it for publication.

## Funding

The study was partially supported by grant Fra2014 to the first author.

### Conflict of interest statement

The authors declare that the research was conducted in the absence of any commercial or financial relationships that could be construed as a potential conflict of interest.

## Footnotes

^1^Traditionally vocabulary was considered a good indicator of intelligence (Binet and Simon, 1905), and in fact vocabulary measures have very high loading on the g-factor (Carroll, 1993; Flanagan and Kaufman, 2004).

^2^The adaptation mainly regarded the type of language used. In particular, in two items the word “expression” instead of the original word “problem” was used, since children in Italian middle school level are more exposed to expression exercises.
